# Development, characterization, and cross-amplification of polymorphic microsatellite markers for North American *Trachymyrmex* and *Mycetomoellerius* ants

**DOI:** 10.1186/s13104-020-05015-3

**Published:** 2020-03-24

**Authors:** Alix E. Matthews, Chase Rowan, Colby Stone, Katrin Kellner, Jon N. Seal

**Affiliations:** Department of Biology, The University of Texas at Tyler, Tyler, TX USA

**Keywords:** Attini, Fungus-gardening ant, Intraspecific genetic diversity, Microsatellites, Population genetics

## Abstract

**Objective:**

The objective of this study is to develop and identify polymorphic microsatellite markers for fungus-gardening (attine) ants in the genus *Trachymyrmex* sensu lato. These ants are important ecosystem engineers and have been a model group for understanding complex symbiotic systems, but very little is understood about the intraspecific genetic patterns across most North American attine species. These microsatellite markers will help to better study intraspecific population genetic structure, gene flow, mating habits, and phylogeographic patterns in these species and potentially other congeners.

**Results:**

Using next-generation sequencing techniques, we identified 17 and 12 polymorphic microsatellite markers from *T. septentrionalis* and *Mycetomoellerius* (formerly *Trachymyrmex*) *turrifex*, respectively, and assessed the genetic diversity of each marker. We also analyzed the cross-amplification success of the *T. septentrionalis* markers in two other closely related *Trachymyrmex* species, and identified 10 and 12 polymorphic markers for *T. arizonensis* and *T. pomonae*, respectively.

## Introduction

Fungus-gardening ants (tribe Attini) have been subject to decades of research elucidating their major evolutionary transitions and radiations [1–5]. Despite these revolutionary advances, most attine research has occurred at higher taxonomic levels (species or higher). Consequently, we know very little about the microevolutionary processes that may underlie macroevolutionary (phylogenetic) patterns. This lack of understanding is partially technical since most genetic markers used to construct phylogenies typically exhibit little intraspecific variation, for both the ants and fungi [2, 6–8]. Mitochondrial DNA (mtDNA) markers, while useful for large scale geographic studies [9–11], may be generally problematic because some attines may have nuclear-incorporated mtDNA-like sequences or whose genomes contain pseudogenes [7, 12]. Moreover, mtDNA is maternally inherited, which may or may not be indicative of intraspecific patterns of gene flow since male and female ants are known to exhibit different patterns of gene flow [13–15]. Finer-level intraspecific examinations have typically required specific diploid markers such as microsatellites or single-nucleotide polymorphisms (SNPs) [16–19]. Both approaches represent co-dominant expression and genome-wide distributions, but microsatellites are short, non-coding, highly variable repetitive motifs [20], whereas SNPs are shorter and found throughout coding and non-coding genomic regions [21].

SNPs are popular in the genomics age because of the large quantities of potentially informative sites quickly generated in a single sequencing run, but they require considerable time and effort to identify and analyze, and are typically restricted to single studies. Conversely, microsatellite markers, once developed, can be used repeatedly in multiple studies, which may reduce their overall expense on a per-project basis [22]. Moreover, recent comparative studies in insects, fungi, and birds reported no major population genetic structural differences between these two approaches [23–26], or that more SNPs were required to produce the same information as a single microsatellite marker [27].

Traditional microsatellite development techniques (i.e., constructing libraries, cloning) have yielded polymorphic microsatellite markers for both higher and lower attines [28–32]. However, because these markers were developed for tropical attines, they may or may not be useful for North American species (J.N. Seal, unpublished observations), which tend to belong to phylogenetically distinct lineages [3, 33]. Next-generation sequencing (NGS) techniques have revolutionized microsatellite marker development in terms of decreased time and cost, and increased quantity of potential markers [34–36], although only a few studies have utilized these techniques for attines. Extensive marker sets exist for *Acromyrmex lundii* [37], *Atta laevigata* [38], and two species in the lower attine genus *Mycocepurus* [18, 39].

Here, our aim was to use NGS techniques to develop microsatellite markers for two additional attine species, *Trachymyrmex septentrionalis* and *Mycetomoellerius turrifex*. Both are common and ecologically important fungus-gardening ants throughout southeastern North America [10, 40, 41]. We also explored the potential for cross-amplification of the *T. septentrionalis* markers in two closely related species: *T. arizonensis* and *T. pomonae*. These three species belong to *Trachymyrmex* sensu stricto, unlike *M. turrifex* which belongs to a new genus, *Mycetomoellerius* [3, 33, 42]. These markers will provide a new tool to study patterns of attine intraspecific genetic variation and phylogeographic structure.

## Main text

### Methods

Primer sets were developed from a single *T. septentrionalis* forager collected in north Florida ([10] Site C; 30.2796, − 84.9004) and a single *M. turrifex* forager collected in central Texas (University of Texas at Austin; 30.2861, − 97.7297). Illumina sequencing and primer design were conducted at the Savannah River Ecology Laboratory (University of Georgia, USA) in the Molecular Ecology Laboratory. A Covaris S220 Focused-ultrasonicator sheared 1 μg of genomic DNA to prepare an Illumina paired-end shotgun library following standard Illumina TruSeq DNA Library Kit protocols and using a multiplex identifier adaptor index. Sequencing was conducted on an Illumina HiSeq 3000 platform (San Diego, CA, USA) producing 100-bp paired-end reads. PAL_FINDER v.0.02.03 [43] was used to analyze five million resulting reads, and those with di-, tri-, tetra-, penta-, and hexanucleotide microsatellites were extracted. Positive reads were batched to a local installation of Primer3 v.2.0.0 [44, 45] for primer design. Markers for which the primer sequence only occurred three or four times in the five million reads were selected to avoid duplication. From this selection of 9293 (*T. septentrionalis*) and 6943 (*M. turrifex*) markers, we chose a subset to test (46 for *T. septentrionalis*; 20 for *M. turrifex*) following an M13-tail PCR protocol [46].

We extracted genomic DNA from individual worker ants (from restricted portions of their respective ranges; Additional file 1) preserved in 95% ethanol at − 20 °C using a QIAamp DNA Micro Kit (QIAGEN). In particular, ants used in the development of *T. septentrionalis* markers were all collected in Florida (i.e., not Texas) (Table 1). For all species, the 10 μL PCR mix contained 1 μL of 10X PCR buffer (1.0X; Applied Biosystems), 1 μL Bioline^®^ dNTP mix (1 mM each; 0.1 mM proportional to total), 1.5 μL of 25 mM MgCl_2_ (3.75 mM; Applied Biosystems), 0.5 μL of 20 μM BSA (1 μM; New England Biolabs), 0.3 μL of 2 μM tag-labeled primer (0.06 μM; forward primer with M13-tail), 0.6 μL of a 2 μM universal dye-labeled primer (0.12 μM; FAM label with M13-tail), 1 μL of 2 μM unlabeled primer (0.2 μM; reverse primer), 0.1 μL of *Taq* polymerase (0.5 U; Applied Biosystems), and 1 μL of DNA template. PCR cycling conditions consisted of an initial denaturation of 4 min at 95 °C, 25 (or 30) cycles of 30 s at 95 °C, 45 s at the primer-specific annealing temperature identified by a temperature gradient program, 45 s at 72 °C, then 8 cycles of 30 s at 95 °C, 45 s at 53 °C, and 45 s at 72 °C, and a final extension of 5 min at 72 °C. PCR products were run on an Applied Biosystems 3730 Genetic Analyzer and fragments were sized with LIZ600 size standard at the University of Texas at Austin DNA Sequencing Facility.

**Table 1:** *Trachymyrmex septentrionalis* polymorphic microsatellite markers analyzed for *Trachymyrmex septentrionalis* ants

Marker	Primer sequence 5′- > 3′		Repeat motif	T_m_ (°C)	Size (bp)	N	K	H_o_	H_e_	PI
Ts3^a,b,c^	F: GCGCGGTTGTTCTTTATGG R: TTGTATTCTTTGTCATACAGTACGTTGC		ATTTT	60.5	264–292	25	12	0.68	0.88	0.03
Ts4^b,c^	F: CTTTGAAATCGTCATCGCGG R: ACGCCCACACGTATACCACC		TCGGC	57.3	242–266	25	11	0.56	0.87	0.03
Ts5^a,c^	F: CCACCTTGGTAACTGTCGCC R: CTTTGAAATCGTCATCGCGG		TCGGC	60.5	230–256	48	11	0.67	0.81	0.06
Ts11^b,c^	F: GCAGATACAAACGTCCTACGTGC R: CGCACATTTGTGACGGACG		TGCG	66.4	274–358	20	13	0.45	0.91	0.02
Ts12^b,c^	F: ATTCCTGGCACGGATACACG R: ACTCTATTGTTGCGCACCGC		ATAC	61.3	164–214	26	9	0.35	0.65	0.16
Ts21^c^	F: CCATCCCAACCATCCTGG R: TTACGATCAGGAGAGCGTGC		AGCC	65.2	302–326	45	11	0.76	0.82	0.05
Ts25^c^	F: CGTAAATTAAAGTGCACAGTCCG R: GATCGCGATTTGGTGATACG		TCTG	61.3	286–346	51	18	0.78	0.86	0.03
Ts32^b,c^	F: ATAACAAGCGGCAGCATCG R: ATTTCGAACTCGCCGGTAGC		TTGG	59.4	200–242	49	15	0.76	0.85	0.04
Ts33	F: AATCAAATGCTTGCGTGTGC R: CCGGTTAGAAGAAACAGGCG		TGCG	60.5	248–296	51	15	0.71	0.73	0.09
Ts34^b,c^	F: GTGAGGGAATGAGAGGGAGG R: TGGTAATGATCGGTACATGATGC		TGCG	61.3	246–490	27	12	0.11	0.81	0.06
Ts35^b,c^	F: TGCTCGATTCGGACACGG R: CTCACAGCGGAGACAAAGGC		ACCG	60.5	188–300	49	14	0.89	0.76	0.08
Ts36^b,c^	F: TGTAGGGATTTAGATGCGGC R: TAGCCAGACCTCGTACTTCG		TGCG	59.4	166–210	13	9	0.15	0.85	0.04
Ts39^c^	F: CTAACAAGATGCGCAGCCC R: TCGAATAATCCAGTCGTGTCG		TGCC	61.3	234–350	50	30	0.84	0.94	0.01
Ts41	F: TTAACGTCGGCATAATTTCGG R: CAATTGACTACGCAGGAGCG		TGCC	61.3	206–236	46	14	0.78	0.82	0.05
Ts43^c^	F: CGTCTTTATATTGTATTTGCTTGATACGC R: GTCCATGCACACGTCCAGC		TGCG	61.3	246–358	47	33	0.72	0.94	0.01
Ts45^b,c^	F: CGTGTCAAGTATGTTCCCGC R: AGTTTCAGGCGCAGGTAGC		TGCG	61.3	174–316	48	28	0.60	0.89	0.02
Ts46^c^	F: GTACGCACATCGTGCTAAACG R: AGCGGTGGTGGTTTCACG		TGCG	60.5	310–348	46	20	0.78	0.92	0.01

Details include: marker information, primer sequences, repeat motif, annealing temperature (T_m_), size range of observed alleles given in base pairs (bp), number of individuals genotyped (N), number of alleles observed (K), observed heterozygosity (H_o_), expected heterozygosity (H_e_), and probability of identity (PI)

^a^Indicates that 30 cycles were used in the PCR profile

^b^Indicates deviation from Hardy–Weinberg expectations after Bonferroni corrections

^c^Indicates the marker shows evidence of null alleles

We scored alleles in Geneious v.10.2.3 [47]. For markers that resulted in high quality PCR product and exhibited polymorphism, we measured genetic variability for each species. We estimated the number of alleles per locus (*K*), observed and expected heterozygosity (H_o_ and H_e_), and the probability of identity (PI; the probability of two independent samples having the same genotype) using GenAlEx v.6.5 [48, 49]. Sample sizes (numbers of individuals tested) for each marker are provided (Tables 1 and 2, Additional files 2 and 3). We assessed deviations from Hardy–Weinberg equilibrium (HWE) and tested for linkage disequilibrium (LD) across all pairs of markers using GENEPOP v.4.2 [50] employing Bonferroni corrections for multiple comparisons. The presence of null alleles was assessed using Microchecker [51].

**Table 2:** *Mycetomoellerius turrifex* polymorphic microsatellite markers analyzed for *Mycetomoellerius turrifex* ants

Marker	Primer sequence 5′- > 3′	Repeat motif	T_m_ (°C)	Size (bp)	N	K	H_o_	H_e_	PI
Tt5	F: ACGGAATGTGTTAACGTGCG R: TATGTGCTCGTCGTTCTCGC	TGCG	60.5	254–322	14	10	0.71	0.86	0.04
Tt10^b,c^	F: ACCGGAGAGCGGTAGAGACC R: TACCCGGCCATTAGAACTCC	AGGT	58.3	286–320	16	11	0.5	0.85	0.04
Tt18	F: GGTGATGGTCGATAGTTTCCG R: ACGACGTATGGGTTCGTGC	AACG	60.5	196–244	16	7	0.44	0.58	0.20
Tt20	F: ATGCAGAGTCAGAGGACCGC R: GAATTGTCTCCACATTATCAAGGG	TGCG	60.5	218–236	15	7	0.67	0.78	0.08
Tt2^b,c^	F: AATGTCGGACGTTTATGGTCG R: GTATCATCGGCACTGCAACG	TTGCG	56.2	266–314	16	7	0.44	0.77	0.09
Tt7^a,b,c^	F: GCAGTATGACTTCTGATCCTTTCG R: CACGTTAATCCAGCACTCCG	TGCG	66.2	156–176	15	4	0.20	0.58	0.26
Tt9^a^	F: ACGCACTGTGTATGTGTGCG R: AGCATATAAGTACGAATAACTGAGATTGG	TGCG	66.2	188–264	10	12	0.70	0.83	0.04
Tt16	F: TCGATTTATTAGAAAGGCTTGCG R: TGCACGAGAGTGTTTGTAGCC	AGTG	55.1	272–288	13	5	0.54	0.74	0.11
Tt6^b,c^	F: TTATATCGATGGCTTCCCACC R: CCCTCTCGATATCTACTCGGTACG	AGGT	57.3	318–358	15	11	0.27	0.85	0.04
Tt14^a^	F: AAGTCGCGTAATGACGATGC R: GAGATATACCTGATTCAACGTCGC	ATAC	57.3	256–378	15	10	0.60	0.77	0.08
Tt15^a,b,c^	F: TGCCTTCATATATGTGCCTTCG R: TGTCGTTAAGAGTTACAGAACAGGG	TGCG	65.3	238–304	12	12	0.42	0.88	0.03
Tt17^b,c^	F: TCACTCAAATCGAATATGTAGATGAGG R: CCCACAGTAATGTCCTAGTAATGTCC	TGCG	64.4	148–198	15	11	0.47	0.88	0.03

Details include: marker information, primer sequences, repeat motif, annealing temperature (T_m_), size range of observed alleles given in base pairs (bp), number of individuals genotyped (N), number of alleles observed (K), observed heterozygosity (H_o_), expected heterozygosity (H_e_), and probability of identity (PI)

^a^Indicates that 30 cycles were used in the PCR profile

^b^Indicates deviation from Hardy–Weinberg expectations after Bonferroni corrections

^c^Indicates the marker shows evidence of null alleles

### Results

For *Trachymyrmex septentrionalis* markers tested on *T. septentrionalis* ants, 37 of the original 46 positively amplified with 17 being reliably amplified and scored. The other 20 either showed weak amplification, weak polymorphism (2–4 alleles per marker), monomorphism, or multi-peak profiles. The number of alleles across all samples of the 17 reliable markers ranged from 9 to 33 (average = 16.2). H_o_ varied from 0.11 to 0.89 (average = 0.62), while H_e_ ranged from 0.65 to 0.94 (average = 0.84). After Bonferroni correction, 9 markers showed significant deviations from HWE. There were two cases of LD detected across the 136 paired markers (between Ts4 and Ts5, and Ts3 and Ts43; Table 1; Additional file 4). Most of the loci tested significantly for the presence of null alleles, which suggests that *T. septentrionalis* may have even higher allelic diversity than what we captured in our sampling (Table 1).

Of the 20 original *Mycetomoellerius turrifex* markers, 17 positively amplified with 12 reliably amplified and scored. The number of alleles across all samples ranged from 4 to 12 (average = 8.9). H_o_ varied from 0.2 to 0.71 (average = 0.49), while H_e_ ranged from 0.58 to 0.88 (average = 0.78). After Bonferroni correction, 6 markers showed significant deviations from HWE. There were no cases of LD detected across the 66 paired markers (Table 2; Additional file 4). In contrast to *T. septentrionalis*, the frequency of null alleles was lower in *M. turrifex* (Table 2).

We tested cross-species amplification of the 46 *T. septentrionalis* markers developed here in both *T. arizonensis* and *T. pomonae*. For *T. arizonensis* (n = 8), 24 markers were positively amplified with 10 reliably scored. Of those 10, all revealed at least 2 alleles, 8 revealed 3 or more alleles, and 9 exhibited some heterozygosity. There were 9 markers in HWE and one violated HWE (Ts38). There was no LD (Additional files 2, 4). For *T. pomonae* (n = 6), 23 markers were successfully amplified and 12 were reliably scored. Of those 12, all revealed at least 2 alleles, 8 revealed 3 or more alleles, and 10 exhibited some heterozygosity. There were 10 in HWE, one violated HWE (also Ts38), and one could not be computed (Ts7) because there was only one copy of the second allele. There was no LD (Additional files 3, 4). Only one of the loci in *T. pomonae* and only two in *T. arizonensis* appeared to have null alleles; this likely results from examining ants collected across a few hectares of pine oak forest (Southwest Research Station) (Additional files 2 and 3).

### Discussion

These newly developed microsatellite markers provide a promising new tool for population genetic, mating, dispersal, and phylogeographic studies of *Trachymyrmex septentrionalis* and *Mycetomoellerius turrifex* populations across their ranges. Positive cross-species amplification also indicates that some of the *T. septentrionalis* markers appear to be conserved in at least two other congeneric species (*T. arizonensis* and *T. pomonae*). This indicates that future analyses for additional individuals across a wider geographic region may be informative for understanding similar questions for *T. arizonensis* and *T. pomonae* and perhaps other closely related *Trachymyrmex*. It also seems likely that the markers developed here for *M. turrifex* should cross amplify in closely related *Mycetomoellerius* species, such as the highly studied *M. zeteki* [42, 52, 53], among others.

While all markers across all four species showed polymorphism/allelic variation, several markers (particularly within *T. septentrionalis*) were hypervariable (over 20 alleles per locus; Table 1). These hypervariable markers, collectively with other polymorphic markers, may be informative for investigating intraspecific dynamics at an especially fine-scale (e.g., mating habits such as multiple-mating or multiple-queen hypotheses by comparing relatedness of individuals within colonies). Despite the extensive polymorphism illustrated by these markers, evidence of null alleles suggests that more alleles may be discovered. The frequency of null alleles may be more pronounced in populations that already demonstrate significant genetic structure [54].

The new markers developed here may also be used in conjunction with mtDNA to better understand dispersal, signatures of population expansion or contraction, and phylogeographic history. For example, male and female ants of several species may mediate gene flow differentially through sex-specific dispersal patterns [13, 14, 55–57], but this has yet been tested in *Trachymyrmex* or *Mycetomoellerius* ants. By employing marker systems with different modes of inheritance, we may be able to discern how ecological dispersal patterns influence ant genetic structure across populations.

In a phylogeographic context, the application of both maternally and biparentally inherited markers may also help to uncover and track the progression of previously unrecognized or poorly understood historical processes such as species origin, divergence, colonization, and genetic drift. For example, the extensive evidence for null alleles in *T. septentrionalis* is likely driven by phylogeographic structure in this species across the relatively restricted range examined in this study (northern Florida) [10]. We explored the 17 *T. septentrionalis* markers across a small subset (total n = 12) of *T. septentrionalis* ants from Texas as compared to a random subset of *T. septentrionalis* ants from Florida. The allelic diversity appears to be much higher in Florida than in Texas (see Additional file 5), which may suggest some fundamental genomic differences in populations east and west of the Mississippi River. These preliminary results align with previous mtDNA results that suggest (1) a significant divergence between populations on either side of the Mississippi River and (2) limited genetic diversity west of the Mississippi River [10, 11]. Future analyses comparing the allelic diversity of populations east and west of the Mississippi River would be insightful for better understanding these patterns.

## Limitations

- The restricted sample size and geographic distribution for *T. arizonensis* and *T. pomonae* may underestimate potential polymorphic markers in these species.
- Markers not deviating from HWE in our initial screenings could potentially deviate in other populations, which may reduce the number of usable markers in certain genetic studies; on the other hand, markers deviating from HWE in the studied populations might not deviate in other populations, which would increase the number of markers.
- The presence of null alleles could be a result of potential phylogeographic structure in the populations studied (especially *T. septentrionalis*), as a result polymorphism may be underestimated.
- Sample sizes per marker for the Texas *T. septentrionalis* subset were smaller compared to Florida, which may limit our interpretations; however, reduced microsatellite diversity would be consistent with lower mtDNA diversity in western populations (Texas).

## Supplementary information

**Additional file 1.** List of individual ant samples used in this study. The table includes individual sample name, host species, state of collection, general sampling location, and coordinates of collection location (unless the property was under private ownership).

**Additional file 2.** Details of the 10 *Trachymyrmex septentrionalis* polymorphic microsatellite markers analyzed for cross-amplification in *Trachymyrmex arizonensis*.

**Additional file 3.** Details of the 12 *Trachymyrmex septentrionalis* polymorphic microsatellite markers analyzed for cross-amplification in *Trachymyrmex pomonae*.

**Additional file 4.** Pairwise comparison results of the linkage disequilibrium analyses for all four species.

**Additional file 5.** Details of the 17 *Trachymyrmex septentrionalis* polymorphic microsatellite markers analyzed for *Trachymyrmex septentrionalis* ants across Texas (TX) and Florida (FL), highlighting the differences between two distant populations. Details include: marker name, size range of observed alleles given in base pairs (bp), number of individuals genotyped (N), number of alleles observed (K).

## Data Availability

The datasets analyzed during the current study are not presently public due to their use in ongoing publication, but the genotypic data for each sample are available upon reasonable request to the corresponding author.

## Abbreviations

bp: Base pair
H_e_: Expected heterozygosity
H_o_: Observed heterozygosity
HWE: Hardy–Weinberg equilibrium
K: Number of alleles
ld: Linkage disequilibrium
mtDNA: Mitochondrial DNA
NGS: Next-generation sequencing
PCR: Polymerase chain reaction
PI: Probability of identity
SNPs: Single-nucleotide polymorphisms
T_m_: Annealing temperature
