# Supplementary material for: Development, characterization, and cross-amplification of polymorphic microsatellite markers for North American Trachymyrmex and Mycetomoellerius ants
Source: BMC Res Notes. 2020 Mar 24;13:173. doi: 10.1186/s13104-020-05015-3 (PMC7092486; doi:10.1186/s13104-020-05015-3)
Supplement: Supplementary file 1 — Additional file 1. List of individual ant samples used in this study. The table includes individual sample name, host species, state of collection, general sampling location, and coordinates of collection location (unless the property was under private ownership). [file 13104_2020_5015_MOESM1_ESM.docx]

Additional file 1. List of individual ants used in this study. The table includes individual sample name, host species, state of collection, general sampling location, and coordinates of collection location (unless the property was under private ownership).

| Individual | Species | | State | | Sampling Location | | Lat | | Lon | |  |  |
| --- | --- | --- | --- | --- | --- | --- | --- | --- | --- | --- | --- | --- |
| SGI46 | | *Trachymyrmex septentrionalis* | | Florida | | St. George Island | | 29.66424 | | -84.862 | |  |
| SGI16 | | *Trachymyrmex septentrionalis* | | Florida | | St. George Island | | 29.66424 | | -84.862 | |  |
| K5 | | *Trachymyrmex septentrionalis* | | Florida | | Apalachicola Ranger District, Apalachicola National Forest | | 30.24058 | | -84.9992 | |  |
| K3 | | *Trachymyrmex septentrionalis* | | Florida | | Apalachicola Ranger District, Apalachicola National Forest | | 30.24058 | | -84.9992 | |  |
| F3 | | *Trachymyrmex septentrionalis* | | Florida | | Wakulla Ranger District, Apalachicola National Forest | | 30.3759 | | -84.3668 | |  |
| F2 | | *Trachymyrmex septentrionalis* | | Florida | | Wakulla Ranger District, Apalachicola National Forest | | 30.3759 | | -84.3668 | |  |
| E3 | | *Trachymyrmex septentrionalis* | | Florida | | Apalachicola Ranger District, Apalachicola National Forest | | 30.20142 | | -84.7282 | |  |
| E2 | | *Trachymyrmex septentrionalis* | | Florida | | Apalachicola Ranger District, Apalachicola National Forest | | 30.20142 | | -84.7282 | |  |
| DLPQ9 | | *Trachymyrmex septentrionalis* | | Florida | | Wakulla Ranger District, Apalachicola National Forest | | 30.3759 | | -84.3668 | |  |
| DLPQ4 | | *Trachymyrmex septentrionalis* | | Florida | | Wakulla Ranger District, Apalachicola National Forest | | 30.3759 | | -84.3668 | |  |
| DLPQ1 | | *Trachymyrmex septentrionalis* | | Florida | | Wakulla Ranger District, Apalachicola National Forest | | 30.3759 | | -84.3668 | |  |
| D5 | | *Trachymyrmex septentrionalis* | | Florida | | Apalachicola Ranger District, Apalachicola National Forest | | 30.27835 | | -84.9156 | |  |
| D4 | | *Trachymyrmex septentrionalis* | | Florida | | Apalachicola Ranger District, Apalachicola National Forest | | 30.27835 | | -84.9156 | |  |
| C7 | | *Trachymyrmex septentrionalis* | | Florida | | Apalachicola Ranger District, Apalachicola National Forest | | 30.2796 | | -84.9004 | |  |
| C6 | | *Trachymyrmex septentrionalis* | | Florida | | Apalachicola Ranger District, Apalachicola National Forest | | 30.2796 | | -84.9004 | |  |
| C5 | | *Trachymyrmex septentrionalis* | | Florida | | Apalachicola Ranger District, Apalachicola National Forest | | 30.2796 | | -84.9004 | |  |
| C3 | | *Trachymyrmex septentrionalis* | | Florida | | Apalachicola Ranger District, Apalachicola National Forest | | 30.2796 | | -84.9004 | |  |
| AP7 | | *Trachymyrmex septentrionalis* | | Florida | | Wakulla Ranger District, Apalachicola National Forest | | 30.35668 | | -84.4969 | |  |
| AP4 | | *Trachymyrmex septentrionalis* | | Florida | | Wakulla Ranger District, Apalachicola National Forest | | 30.35668 | | -84.4969 | |  |
| AP2 | | *Trachymyrmex septentrionalis* | | Florida | | Wakulla Ranger District, Apalachicola National Forest | | 30.35668 | | -84.4969 | |  |
| ANFL13 | | *Trachymyrmex septentrionalis* | | Florida | | Apalachicola Ranger District, Apalachicola National Forest | | 30.20419 | | -84.9217 | |  |
| ANFJ35 | | *Trachymyrmex septentrionalis* | | Florida | | Apalachicola Ranger District, Apalachicola National Forest | | 30.16626 | | -85.0152 | |  |
| ANFH25 | | *Trachymyrmex septentrionalis* | | Florida | | Apalachicola Ranger District, Apalachicola National Forest | | 30.1645 | | -84.9929 | |  |
| ANFG14 | | *Trachymyrmex septentrionalis* | | Florida | | Apalachicola Ranger District, Apalachicola National Forest | | 29.99765 | | -84.9792 | |  |
| ANF15 | | *Trachymyrmex septentrionalis* | | Florida | | Wakulla Ranger District, Apalachicola National Forest | | 30.3795 | | -84.335 | |  |
| ANF14 | | *Trachymyrmex septentrionalis* | | Florida | | Wakulla Ranger District, Apalachicola National Forest | | 30.3795 | | -84.335 | |  |
| ANF13 | | *Trachymyrmex septentrionalis* | | Florida | | Wakulla Ranger District, Apalachicola National Forest | | 30.3795 | | -84.335 | |  |
| ANF9 | | *Trachymyrmex septentrionalis* | | Florida | | Wakulla Ranger District, Apalachicola National Forest | | 30.3795 | | -84.335 | |  |
| ANF8 | | *Trachymyrmex septentrionalis* | | Florida | | Wakulla Ranger District, Apalachicola National Forest | | 30.3795 | | -84.335 | |  |
| ANF6 | | *Trachymyrmex septentrionalis* | | Florida | | Wakulla Ranger District, Apalachicola National Forest | | 30.3795 | | -84.335 | |  |
| 25ANF1 | | *Trachymyrmex septentrionalis* | | Florida | | Wakulla Ranger District, Apalachicola National Forest | | 30.3795 | | -84.335 | |  |
| UCF5 | | *Trachymyrmex septentrionalis* | | Florida | | University of Central Florida | | 28.58856 | | -81.2027 | |  |
| SGI66 | | *Trachymyrmex septentrionalis* | | Florida | | St. George Island | | 29.66424 | | -84.862 | |  |
| SGI56 | | *Trachymyrmex septentrionalis* | | Florida | | St. George Island | | 29.66424 | | -84.862 | |  |
| SGI36 | | *Trachymyrmex septentrionalis* | | Florida | | St. George Island | | 29.66424 | | -84.862 | |  |
| SGI26 | | *Trachymyrmex septentrionalis* | | Florida | | St. George Island | | 29.66424 | | -84.862 | |  |
| E4 | | *Trachymyrmex septentrionalis* | | Florida | | Apalachicola Ranger District, Apalachicola National Forest | | 30.20142 | | -84.7282 | |  |
| E1 | | *Trachymyrmex septentrionalis* | | Florida | | Apalachicola Ranger District, Apalachicola National Forest | | 30.20142 | | -84.7282 | |  |
| D3 | | *Trachymyrmex septentrionalis* | | Florida | | Apalachicola Ranger District, Apalachicola National Forest | | 30.27835 | | -84.9156 | |  |
| D2 | | *Trachymyrmex septentrionalis* | | Florida | | Apalachicola Ranger District, Apalachicola National Forest | | 30.27835 | | -84.9156 | |  |
| Chuluota3 | | *Trachymyrmex septentrionalis* | | Florida | | Chuluota | | 28.61796 | | -81.05 | |  |
| Chuluota1 | | *Trachymyrmex septentrionalis* | | Florida | | Chuluota | | 28.61796 | | -81.05 | |  |
| AP6 | | *Trachymyrmex septentrionalis* | | Florida | | Wakulla Ranger District, Apalachicola National Forest | | 30.35668 | | -84.4969 | |  |
| AP3 | | *Trachymyrmex septentrionalis* | | Florida | | Wakulla Ranger District, Apalachicola National Forest | | 30.35668 | | -84.4969 | |  |
| ANFL33 | | *Trachymyrmex septentrionalis* | | Florida | | Apalachicola Ranger District, Apalachicola National Forest | | 30.20419 | | -84.9217 | |  |
| ANFL23 | | *Trachymyrmex septentrionalis* | | Florida | | Apalachicola Ranger District, Apalachicola National Forest | | 30.20419 | | -84.9217 | |  |
| ANFK213 | | *Trachymyrmex septentrionalis* | | Florida | | Apalachicola Ranger District, Apalachicola National Forest | | 30.24058 | | -84.9992 | |  |
| ANFK33 | | *Trachymyrmex septentrionalis* | | Florida | | Apalachicola Ranger District, Apalachicola National Forest | | 30.24058 | | -84.9992 | |  |
| ANFK13 | | *Trachymyrmex septentrionalis* | | Florida | | Apalachicola Ranger District, Apalachicola National Forest | | 30.24058 | | -84.9992 | |  |
| ANFJ55 | | *Trachymyrmex septentrionalis* | | Florida | | Apalachicola Ranger District, Apalachicola National Forest | | 30.16626 | | -85.0152 | |  |
| ANFJ25 | | *Trachymyrmex septentrionalis* | | Florida | | Apalachicola Ranger District, Apalachicola National Forest | | 30.16626 | | -85.0152 | |  |
| ANFH45 | | *Trachymyrmex septentrionalis* | | Florida | | Apalachicola Ranger District, Apalachicola National Forest | | 30.1645 | | -84.9929 | |  |
| ANFH15 | | *Trachymyrmex septentrionalis* | | Florida | | Apalachicola Ranger District, Apalachicola National Forest | | 30.1645 | | -84.9929 | |  |
| ANFG44 | | *Trachymyrmex septentrionalis* | | Florida | | Apalachicola Ranger District, Apalachicola National Forest | | 29.99765 | | -84.9792 | |  |
| ANFG34 | | *Trachymyrmex septentrionalis* | | Florida | | Apalachicola Ranger District, Apalachicola National Forest | | 29.99765 | | -84.9792 | |  |
| Lucky1 | | *Trachymyrmex septentrionalis* | | Florida | | Ordway-Swisher Biological Station | | 29.69359 | | -82.0234 | |  |
| Lucky2 | | *Trachymyrmex septentrionalis* | | Florida | | Ordway-Swisher Biological Station | | 29.69359 | | -82.0234 | |  |
| Lucky3 | | *Trachymyrmex septentrionalis* | | Florida | | Ordway-Swisher Biological Station | | 29.69359 | | -82.0234 | |  |
| Lucky4 | | *Trachymyrmex septentrionalis* | | Florida | | Ordway-Swisher Biological Station | | 29.69359 | | -82.0234 | |  |
| Lucky5 | | *Trachymyrmex septentrionalis* | | Florida | | University of Florida | | 29.61027 | | -82.3643 | |  |
| Lucky6 | | *Trachymyrmex septentrionalis* | | Florida | | Ordway-Swisher Biological Station | | 29.69359 | | -82.0234 | |  |
| Lucky7 | | *Trachymyrmex septentrionalis* | | Florida | | Ordway-Swisher Biological Station | | 29.69359 | | -82.0234 | |  |
| Lucky8 | | *Trachymyrmex septentrionalis* | | Florida | | University of Florida | | 29.63557 | | -82.3597 | |  |
| Lucky9 | | *Trachymyrmex septentrionalis* | | Florida | | University of Florida | | 29.64192 | | -82.3557 | |  |
| Lucky10 | | *Trachymyrmex septentrionalis* | | Florida | | Ordway-Swisher Biological Station | | 29.69359 | | -82.0234 | |  |
| Lucky11 | | *Trachymyrmex septentrionalis* | | Florida | | Ordway-Swisher Biological Station | | 29.69359 | | -82.0234 | |  |
| UCF1 2018 | | *Trachymyrmex septentrionalis* | | Florida | | University of Central Florida | | 28.60379 | | -81.1931 | |  |
| UCF2 2018 | | *Trachymyrmex septentrionalis* | | Florida | | University of Central Florida | | 28.60379 | | -81.1931 | |  |
| UCF3 2018 | | *Trachymyrmex septentrionalis* | | Florida | | University of Central Florida | | 28.60379 | | -81.1931 | |  |
| UCF4 2018 | | *Trachymyrmex septentrionalis* | | Florida | | University of Central Florida | | 28.60379 | | -81.1931 | |  |
| S17 | | *Trachymyrmex septentrionalis* | | Texas | | Stengl Lost Pines Biological Station | | 30.0873 | | -97.1736 | |  |
| S3 | | *Trachymyrmex septentrionalis* | | Texas | | Stengl Lost Pines Biological Station | | 30.0873 | | -97.1736 | |  |
| 24SS170529-1 | | *Trachymyrmex septentrionalis* | | Texas | | The University of Texas at Tyler | | 32.3131 | | -95.2546 | |  |
| 23SS170407-1 | | *Trachymyrmex septentrionalis* | | Texas | | Stengl Lost Pines Biological Station | | 30.0873 | | -97.1736 | |  |
| 22JNS170407-4 | | *Trachymyrmex septentrionalis* | | Texas | | Stengl Lost Pines Biological Station | | 30.0873 | | -97.1736 | |  |
| 21JNS170407-2 | | *Trachymyrmex septentrionalis* | | Texas | | Stengl Lost Pines Biological Station | | 30.0873 | | -97.1736 | |  |
| 3Cald3 | | *Trachymyrmex septentrionalis* | | Texas | | Caldwell Co. | | Private | |  | |  |
| 4Cald4 | | *Trachymyrmex septentrionalis* | | Texas | | Caldwell Co. | | Private | |  | | |
| Bastrop9-2 | | *Trachymyrmex septentrionalis* | | Texas | | Bastrop Co. | | Private | |  | |  |
| Bastrop8-5 | | *Trachymyrmex septentrionalis* | | Texas | | Bastop Co. | | Private | |  | |  |
| S14 | | *Trachymyrmex septentrionalis* | | Texas | | Stengl Lost Pines Biological Station | | 30.0873 | | -97.1736 | |  |
| S2 | | *Trachymyrmex septentrionalis* | | Texas | | Stengl Lost Pines Biological Station | | 30.0873 | | -97.1736 | |  |
| BenstenSP1 | | *Mycetomoellerius turrifex* | | Texas | | Bentsen State Park | | 26.18534 | | -98.3795 | |  |
| BenstenSP2 | | *Mycetomoellerius turrifex* | | Texas | | Bentsen State Park | | 26.18534 | | -98.3795 | |  |
| BenstenSP3 | | *Mycetomoellerius turrifex* | | Texas | | Bentsen State Park | | 26.18534 | | -98.3795 | |  |
| BenstenSP4 | | *Mycetomoellerius turrifex* | | Texas | | Bentsen State Park | | 26.18534 | | -98.3795 | |  |
| BenstenSP5 | | *Mycetomoellerius turrifex* | | Texas | | Bentsen State Park | | 26.18534 | | -98.3795 | |  |
| JLL170514-3Tt | | *Mycetomoellerius turrifex* | | Texas | | Waller Co. | | Private | | | |  |
| JLL180511-1Tt | | *Mycetomoellerius turrifex* | | Texas | | The University of Texas at Tyler | | 32.3131 | | -95.2546 | |  |
| JLL180513-2Tt | | *Mycetomoellerius turrifex* | | Texas | | Brackenridge Field Laboratory | | 30.28302 | | -97.7793 | |  |
| JLL180513-4Tt | | *Mycetomoellerius turrifex* | | Texas | | Brackenridge Field Laboratory | | 30.28302 | | -97.7793 | |  |
| JLL180516-4Tt | | *Mycetomoellerius turrifex* | | Texas | | Henderson Co. | | Private | | | |  |
| JNS170406-Tt3 | | *Mycetomoellerius turrifex* | | Texas | | Stengl Lost Pines Biological Station | | 30.0873 | | -97.1736 | |  |
| JNS170407-3Tt | | *Mycetomoellerius turrifex* | | Texas | | Stengl Lost Pines Biological Station | | 30.0873 | | -97.1736 | |  |
| JNS170407-Tt2 | | *Mycetomoellerius turrifex* | | Texas | | Stengl Lost Pines Biological Station | | 30.0873 | | -97.1736 | |  |
| JNS170531-1Tt | | *Mycetomoellerius turrifex* | | Texas | | The University of Texas at Tyler | | 32.3131 | | -95.2546 | |  |
| LBJOak | | *Mycetomoellerius turrifex* | | Texas | | The University of Texas at Austin | | 30.28612 | | -97.7297 | |  |
| Tyler3 | | *Mycetomoellerius turrifex* | | Texas | | Tyler Co. | | Private | | | |  |
| SS180712-3Ta | | *Trachymyrmex arizonensis* | | Arizona | | Southwestern Research Station | | 31.88403 | | -109.208 | |  |
| JNS180713-1Ta | | *Trachymyrmex arizonensis* | | Arizona | | Southwestern Research Station | | 31.88245 | | -109.209 | |  |
| SS180714-1Ta | | *Trachymyrmex arizonensis* | | Arizona | | Southwestern Research Station | | 31.88255 | | -109.209 | |  |
| KB180715-1Ta | | *Trachymyrmex arizonensis* | | Arizona | | Southwestern Research Station | | 31.88369 | | -109.209 | |  |
| KB180718-2Ta | | *Trachymyrmex arizonensis* | | Arizona | | Southwestern Research Station | | 31.88572 | | -109.207 | |  |
| KB180718-3Ta | | *Trachymyrmex arizonensis* | | Arizona | | Southwestern Research Station | | 31.8858 | | -109.207 | |  |
| SS180718-1Ta | | *Trachymyrmex arizonensis* | | Arizona | | Southwestern Research Station | | 31.8848 | | -109.206 | |  |
| SS180721-1Ta | | *Trachymyrmex arizonensis* | | Arizona | | Southwestern Research Station | | 31.89745 | | -109.218 | |  |
| JNS180714-1Tp | | *Trachymyrmex pomonae* | | Arizona | | Southwestern Research Station | | 31.88472 | | -109.206 | |  |
| SS180717-2Tp | | *Trachymyrmex pomonae* | | Arizona | | Southwestern Research Station | | 31.88496 | | -109.206 | |  |
| KB180716-1Tp | | *Trachymyrmex pomonae* | | Arizona | | Southwestern Research Station | | 31.88493 | | -109.206 | |  |
| KB180717-1Tp | | *Trachymyrmex pomonae* | | Arizona | | Southwestern Research Station | | 31.88491 | | -109.206 | |  |
| JNS180714-2Tp | | *Trachymyrmex pomonae* | | Arizona | | Southwestern Research Station | | 31.88471 | | -109.206 | |  |
| KB180716-2Tp | | *Trachymyrmex pomonae* | | Arizona | | Southwestern Research Station | | 31.88479 | | -109.206 | |  |
