# Supplementary material for: Development, characterization, and cross-amplification of polymorphic microsatellite markers for North American Trachymyrmex and Mycetomoellerius ants
Source: BMC Res Notes. 2020 Mar 24;13:173. doi: 10.1186/s13104-020-05015-3 (PMC7092486; doi:10.1186/s13104-020-05015-3)
Supplement: Supplementary file 2 — Additional file 2. Details of the 10 Trachymyrmex septentrionalis polymorphic microsatellite markers analyzed for cross-amplification in Trachymyrmex arizonensis. [file 13104_2020_5015_MOESM2_ESM.docx]

Additional file 2. *Trachymyrmex septentrionalis* polymorphic microsatellite markers analyzed for *Trachymyrmex arizonensis* ants/cross-amplification. Details include: marker information, primer sequences, repeat motif, annealing temperature (T_m_), size range of observed alleles given in base pairs (bp), number of individuals genotyped (N), number of alleles observed (K), observed heterozygosity (H_o_), expected heterozygosity (H_e_), and probability of identity (PI). Superscript ^a^ indicates deviation from Hardy-Weinberg expectations after Bonferroni corrections, and superscript ^b^ indicates the marker shows evidence of null alleles.

| Marker | Primer sequence 5' -> 3' | Repeat motif | T_m_ (°C) | Size (bp) | N | K | H_o_ | H_e_ | PI |
| --- | --- | --- | --- | --- | --- | --- | --- | --- | --- |
| Ts11 | F: GCAGATACAAACGTCCTACGTGC | TGCG | 66.4 | 266-282 | 8 | 3 | 0.13 | 0.23 | 0.61 |
|  | R: CGCACATTTGTGACGGACG |  |  |  |  |  |  |  |  |
| Ts12 | F: ATTCCTGGCACGGATACACG | ATAC | 61.3 | 162-168 | 8 | 4 | 0.50 | 0.48 | 0.31 |
|  | R: ACTCTATTGTTGCGCACCGC |  |  |  |  |  |  |  |  |
| Ts33 | F: AATCAAATGCTTGCGTGTGC | TGCG | 60.5 | 264-284 | 8 | 2 | 0.50 | 0.50 | 0.38 |
|  | R: CCGGTTAGAAGAAACAGGCG |  |  |  |  |  |  |  |  |
| Ts35 | F: TGCTCGATTCGGACACGG | ACCG | 60.5 | 268-280 | 8 | 3 | 0.63 | 0.48 | 0.32 |
|  | R: CTCACAGCGGAGACAAAGGC |  |  |  |  |  |  |  |  |
| Ts38 ^a,b^ | F: AGACTGCTGGCTACGCTCG | ATAC | 60.5 | 274-282 | 8 | 5 | 0.00 | 0.75 | 0.10 |
|  | R: CGTGGTGACACTCTCATTTCG |  |  |  |  |  |  |  |  |
| Ts39 ^b^ | F: CTAACAAGATGCGCAGCCC | TGCG | 61.3 | 236-256 | 8 | 4 | 0.25 | 0.61 | 0.20 |
|  | R: TCGAATAATCCAGTCGTGTCG |  |  |  |  |  |  |  |  |
| Ts4 | F: CTTTGAAATCGTCATCGCGG | TCGGC | 57.3 | 246-260 | 8 | 3 | 0.38 | 0.54 | 0.31 |
|  | R: ACGCCCACACGTATACCACC |  |  |  |  |  |  |  |  |
| Ts41 | F: TTAACGTCGGCATAATTTCGG | TGCC | 61.3 | 200-212 | 6 | 3 | 0.50 | 0.54 | 0.29 |
|  | R: CAATTGACTACGCAGGAGCG |  |  |  |  |  |  |  |  |
| Ts44 | F: GCGCGAAATTGAAGAGTAAGC | TGCC | 60.5 | 308-320 | 8 | 3 | 0.63 | 0.59 | 0.25 |
|  | R: GCGAACGATCGAGTATGACG |  |  |  |  |  |  |  |  |
| Ts45 | F: CGTGTCAAGTATGTTCCCGC | TGCG | 61.3 | 162-168 | 8 | 2 | 0.75 | 0.47 | 0.39 |
|  | R: AGTTTCAGGCGCAGGTAGC |  |  |  |  |  |  |  |  |
