# Supplementary material for: Development, characterization, and cross-amplification of polymorphic microsatellite markers for North American Trachymyrmex and Mycetomoellerius ants
Source: BMC Res Notes. 2020 Mar 24;13:173. doi: 10.1186/s13104-020-05015-3 (PMC7092486; doi:10.1186/s13104-020-05015-3)
Supplement: Supplementary file 3 — Additional file 3. Details of the 12 Trachymyrmex septentrionalis polymorphic microsatellite markers analyzed for cross-amplification in Trachymyrmex pomonae. [file 13104_2020_5015_MOESM3_ESM.docx]

Additional file 3. *Trachymyrmex septentrionalis* polymorphic microsatellite markers analyzed for *Trachymyrmex pomonae* ants/cross-amplification. Details include: marker information, primer sequences, repeat motif, annealing temperature (T_m_), size range of observed alleles given in base pairs (bp), number of individuals genotyped (N), number of alleles observed (K), observed heterozygosity (H_o_), expected heterozygosity (H_e_), and probability of identity (PI). Superscript ^a^ indicates deviation from Hardy-Weinberg expectations after Bonferroni corrections, superscript ^b^ indicates no Hardy-Weinberg information, and superscript ^c^ indicates the marker shows evidence of null alleles.

| Marker | Primer sequence 5' -> 3' | Repeat motif | T_m_ (°C) | Size (bp) | N | K | H_o_ | H_e_ | PI |
| --- | --- | --- | --- | --- | --- | --- | --- | --- | --- |
| Ts11 | F: GCAGATACAAACGTCCTACGTGC | TGCG | 66.4 | 278-294 | 6 | 2 | 0.83 | 0.49 | 0.38 |
|  | R: CGCACATTTGTGACGGACG | | |  |  |  |  |  |  |
| Ts13 | F: CGAGAGATAACGGACGTTGC | AACG | 64 | 432-452 | 6 | 3 | 0.67 | 0.49 | 0.33 |
|  | R: ACGTGTGTTCATTCGTTGCC | | |  |  |  |  |  |  |
| Ts21 | F: CCATCCCAACCATCCTGG | AGCC | 65.2 | 300-308 | 6 | 3 | 0.33 | 0.49 | 0.33 |
|  | R: TTACGATCAGGAGAGCGTGC | | |  |  |  |  |  |  |
| Ts32 | F: ATAACAAGCGGCAGCATCG | TTGG | 59.4 | 206-218 | 6 | 3 | 0.83 | 0.57 | 0.25 |
|  | R: ATTTCGAACTCGCCGGTAGC | | |  |  |  |  |  |  |
| Ts35 | F: TGCTCGATTCGGACACGG | ACCG | 60.5 | 196-288 | 6 | 3 | 1.00 | 0.57 | 0.28 |
|  | R: CTCACAGCGGAGACAAAGGC | | |  |  |  |  |  |  |
| Ts38 ^a,b^ | F: AGACTGCTGGCTACGCTCG | ATAC | 60.5 | 258-270 | 6 | 3 | 0.00 | 0.67 | 0.19 |
|  | R: CGTGGTGACACTCTCATTTCG | | |  |  |  |  |  |  |
| Ts39 | F: CTAACAAGATGCGCAGCCC | TGCG | 61.3 | 238-250 | 6 | 3 | 0.17 | 0.40 | 0.40 |
|  | R: TCGAATAATCCAGTCGTGTCG | | |  |  |  |  |  |  |
| Ts41 | F: TTAACGTCGGCATAATTTCGG | TGCC | 61.3 | 194-196 | 5 | 2 | 0.00 | 0.48 | 0.39 |
|  | R: CAATTGACTACGCAGGAGCG | | |  |  |  |  |  |  |
| Ts44 | F: GCGCGAAATTGAAGAGTAAGC | TGCC | 60.5 | 256-304 | 6 | 2 | 0.17 | 0.38 | 0.46 |
|  | R: GCGAACGATCGAGTATGACG | | |  |  |  |  |  |  |
| Ts45 | F: CGTGTCAAGTATGTTCCCGC | TGCG | 61.3 | 166-202 | 6 | 3 | 0.17 | 0.40 | 0.40 |
|  | R: AGTTTCAGGCGCAGGTAGC | | |  |  |  |  |  |  |
| Ts46 | F: GTACGCACATCGTGCTAAACG | ACCG | 60.5 | 314-338 | 6 | 4 | 0.67 | 0.65 | 0.17 |
|  | R: AGCGGTGGTGGTTTCACG | | |  |  |  |  |  |  |
| Ts7 ^b^ | F: GAACTTCCCTCCCTCGAACC | TGCG | 61.3 | 198-202 | 6 | 2 | 0.17 | 0.15 | 0.73 |
|  | R: TGAACGTGTAAAGATTGCATACAGC | | | |  |  |  |  |  |
