# Supplementary material for: Development, characterization, and cross-amplification of polymorphic microsatellite markers for North American Trachymyrmex and Mycetomoellerius ants
Source: BMC Res Notes. 2020 Mar 24;13:173. doi: 10.1186/s13104-020-05015-3 (PMC7092486; doi:10.1186/s13104-020-05015-3)
Supplement: Supplementary file 4 — Additional file 4. Pairwise comparison results of the linkage disequilibrium analyses for all four species. [file 13104_2020_5015_MOESM4_ESM.docx]

Additional file 4. Pairwise comparisons and associated P-values of the linkage disequilibrium analyses for all four species. Linkage disequilibrium was only found in two cases for *Trachymyrmex septentrionalis*: between Ts4 and Ts5, and between Ts3 and Ts43 (i.e., with alpha level of significance set at 0.05 for 136 pairwise tests, the Bonferroni-corrected alpha is 0.00037), and are denoted with an asterisks (*).

| **Species** | **Locus #1** | **Locus #2** | **P-value** | **S.E.** |
| --- | --- | --- | --- | --- |
| *Trachymyrmex septentrionalis* | Ts11 | Ts12 | 0.767085 | 0.007867 |
| *Trachymyrmex septentrionalis* | Ts11 | Ts21 | 1 | 0 |
| *Trachymyrmex septentrionalis* | Ts12 | Ts21 | 0.789036 | 0.006691 |
| *Trachymyrmex septentrionalis* | Ts11 | Ts25 | 1 | 0 |
| *Trachymyrmex septentrionalis* | Ts12 | Ts25 | 0.697881 | 0.012661 |
| *Trachymyrmex septentrionalis* | Ts21 | Ts25 | 0.81362 | 0.019886 |
| *Trachymyrmex septentrionalis* | Ts11 | Ts3 | 0.209096 | 0.016981 |
| *Trachymyrmex septentrionalis* | Ts12 | Ts3 | 0.28647 | 0.014687 |
| *Trachymyrmex septentrionalis* | Ts21 | Ts3 | 1 | 0 |
| *Trachymyrmex septentrionalis* | Ts25 | Ts3 | 1 | 0 |
| *Trachymyrmex septentrionalis* | Ts11 | Ts32 | 0.093167 | 0.01267 |
| *Trachymyrmex septentrionalis* | Ts12 | Ts32 | 0.116071 | 0.010164 |
| *Trachymyrmex septentrionalis* | Ts21 | Ts32 | 0.758806 | 0.02305 |
| *Trachymyrmex septentrionalis* | Ts25 | Ts32 | 0.28427 | 0.028717 |
| *Trachymyrmex septentrionalis* | Ts3 | Ts32 | 0.021718 | 0.005818 |
| *Trachymyrmex septentrionalis* | Ts11 | Ts33 | 0.022352 | 0.004038 |
| *Trachymyrmex septentrionalis* | Ts12 | Ts33 | 0.812089 | 0.009088 |
| *Trachymyrmex septentrionalis* | Ts21 | Ts33 | 0.319104 | 0.02064 |
| *Trachymyrmex septentrionalis* | Ts25 | Ts33 | 0.024081 | 0.008955 |
| *Trachymyrmex septentrionalis* | Ts3 | Ts33 | 0.688258 | 0.017654 |
| *Trachymyrmex septentrionalis* | Ts32 | Ts33 | 0.309484 | 0.027384 |
| *Trachymyrmex septentrionalis* | Ts11 | Ts34 | 1 | 0 |
| *Trachymyrmex septentrionalis* | Ts12 | Ts34 | 0.003354 | 0.000687 |
| *Trachymyrmex septentrionalis* | Ts21 | Ts34 | 0.317256 | 0.009804 |
| *Trachymyrmex septentrionalis* | Ts25 | Ts34 | 0.915572 | 0.005607 |
| *Trachymyrmex septentrionalis* | Ts3 | Ts34 | 0.420202 | 0.011963 |
| *Trachymyrmex septentrionalis* | Ts32 | Ts34 | 0.337661 | 0.011756 |
| *Trachymyrmex septentrionalis* | Ts33 | Ts34 | 0.877028 | 0.008109 |
| *Trachymyrmex septentrionalis* | Ts11 | Ts35 | 0.292865 | 0.01494 |
| *Trachymyrmex septentrionalis* | Ts12 | Ts35 | 0.074947 | 0.005638 |
| *Trachymyrmex septentrionalis* | Ts21 | Ts35 | 0.754015 | 0.01481 |
| *Trachymyrmex septentrionalis* | Ts25 | Ts35 | 0.691562 | 0.022916 |
| *Trachymyrmex septentrionalis* | Ts3 | Ts35 | 0.697529 | 0.01581 |
| *Trachymyrmex septentrionalis* | Ts32 | Ts35 | 0.05412 | 0.012454 |
| *Trachymyrmex septentrionalis* | Ts33 | Ts35 | 0.085807 | 0.013193 |
| *Trachymyrmex septentrionalis* | Ts34 | Ts35 | 0.920363 | 0.005937 |
| *Trachymyrmex septentrionalis* | Ts11 | Ts36 | No information | |
| *Trachymyrmex septentrionalis* | Ts12 | Ts36 | 1 | 0 |
| *Trachymyrmex septentrionalis* | Ts21 | Ts36 | 1 | 0 |
| *Trachymyrmex septentrionalis* | Ts25 | Ts36 | 0.212911 | 0.005554 |
| *Trachymyrmex septentrionalis* | Ts3 | Ts36 | 1 | 0 |
| *Trachymyrmex septentrionalis* | Ts32 | Ts36 | 1 | 0 |
| *Trachymyrmex septentrionalis* | Ts33 | Ts36 | 1 | 0 |
| *Trachymyrmex septentrionalis* | Ts34 | Ts36 | 0.490403 | 0.006157 |
| *Trachymyrmex septentrionalis* | Ts35 | Ts36 | 0.040482 | 0.003367 |
| *Trachymyrmex septentrionalis* | Ts11 | Ts39 | 1 | 0 |
| *Trachymyrmex septentrionalis* | Ts12 | Ts39 | 0.579899 | 0.015794 |
| *Trachymyrmex septentrionalis* | Ts21 | Ts39 | 0.541944 | 0.027907 |
| *Trachymyrmex septentrionalis* | Ts25 | Ts39 | 1 | 0 |
| *Trachymyrmex septentrionalis* | Ts3 | Ts39 | 1 | 0 |
| *Trachymyrmex septentrionalis* | Ts32 | Ts39 | 0.347008 | 0.03105 |
| *Trachymyrmex septentrionalis* | Ts33 | Ts39 | 0.031105 | 0.009745 |
| *Trachymyrmex septentrionalis* | Ts34 | Ts39 | 0.197712 | 0.013653 |
| *Trachymyrmex septentrionalis* | Ts35 | Ts39 | 0.841558 | 0.018815 |
| *Trachymyrmex septentrionalis* | Ts36 | Ts39 | 0.330284 | 0.008453 |
| *Trachymyrmex septentrionalis* | Ts11 | Ts4 | 1 | 0 |
| *Trachymyrmex septentrionalis* | Ts12 | Ts4 | 0.120424 | 0.011875 |
| *Trachymyrmex septentrionalis* | Ts21 | Ts4 | 1 | 0 |
| *Trachymyrmex septentrionalis* | Ts25 | Ts4 | 1 | 0 |
| *Trachymyrmex septentrionalis* | Ts3 | Ts4 | 0.225552 | 0.020981 |
| *Trachymyrmex septentrionalis* | Ts32 | Ts4 | 1 | 0 |
| *Trachymyrmex septentrionalis* | Ts33 | Ts4 | 1 | 0 |
| *Trachymyrmex septentrionalis* | Ts34 | Ts4 | 0.445835 | 0.012539 |
| *Trachymyrmex septentrionalis* | Ts35 | Ts4 | 1 | 0 |
| *Trachymyrmex septentrionalis* | Ts36 | Ts4 | 1 | 0 |
| *Trachymyrmex septentrionalis* | Ts39 | Ts4 | 1 | 0 |
| *Trachymyrmex septentrionalis* | Ts11 | Ts41 | 1 | 0 |
| *Trachymyrmex septentrionalis* | Ts12 | Ts41 | 0.987909 | 0.001912 |
| *Trachymyrmex septentrionalis* | Ts21 | Ts41 | 0.687425 | 0.022925 |
| *Trachymyrmex septentrionalis* | Ts25 | Ts41 | 0.702231 | 0.027819 |
| *Trachymyrmex septentrionalis* | Ts3 | Ts41 | 0.500732 | 0.022221 |
| *Trachymyrmex septentrionalis* | Ts32 | Ts41 | 0.301239 | 0.027246 |
| *Trachymyrmex septentrionalis* | Ts33 | Ts41 | 0.10889 | 0.018067 |
| *Trachymyrmex septentrionalis* | Ts34 | Ts41 | 0.704398 | 0.011958 |
| *Trachymyrmex septentrionalis* | Ts35 | Ts41 | 0.528925 | 0.024054 |
| *Trachymyrmex septentrionalis* | Ts36 | Ts41 | 1 | 0 |
| *Trachymyrmex septentrionalis* | Ts39 | Ts41 | 1 | 0 |
| *Trachymyrmex septentrionalis* | Ts4 | Ts41 | 0.401906 | 0.021018 |
| *Trachymyrmex septentrionalis* | Ts11 | Ts43 | 1 | 0 |
| *Trachymyrmex septentrionalis* | Ts12 | Ts43 | 0.180442 | 0.010325 |
| *Trachymyrmex septentrionalis* | Ts21 | Ts43 | 1 | 0 |
| *Trachymyrmex septentrionalis* | Ts25 | Ts43 | 1 | 0 |
| *Trachymyrmex septentrionalis* | Ts3 | Ts43 | 0.0003* | 0.0003 |
| *Trachymyrmex septentrionalis* | Ts32 | Ts43 | 0.011372 | 0.0067 |
| *Trachymyrmex septentrionalis* | Ts33 | Ts43 | 0.009514 | 0.004856 |
| *Trachymyrmex septentrionalis* | Ts34 | Ts43 | 0.507047 | 0.018232 |
| *Trachymyrmex septentrionalis* | Ts35 | Ts43 | 0.744885 | 0.025422 |
| *Trachymyrmex septentrionalis* | Ts36 | Ts43 | 1 | 0 |
| *Trachymyrmex septentrionalis* | Ts39 | Ts43 | 0.001031 | 0.001031 |
| *Trachymyrmex septentrionalis* | Ts4 | Ts43 | 0.09786 | 0.014716 |
| *Trachymyrmex septentrionalis* | Ts41 | Ts43 | 0.378463 | 0.031725 |
| *Trachymyrmex septentrionalis* | Ts11 | Ts45 | 0.127337 | 0.012788 |
| *Trachymyrmex septentrionalis* | Ts12 | Ts45 | 0.324521 | 0.013576 |
| *Trachymyrmex septentrionalis* | Ts21 | Ts45 | 0.372834 | 0.022126 |
| *Trachymyrmex septentrionalis* | Ts25 | Ts45 | 1 | 0 |
| *Trachymyrmex septentrionalis* | Ts3 | Ts45 | 1 | 0 |
| *Trachymyrmex septentrionalis* | Ts32 | Ts45 | 0.533264 | 0.026816 |
| *Trachymyrmex septentrionalis* | Ts33 | Ts45 | 0.993173 | 0.003493 |
| *Trachymyrmex septentrionalis* | Ts34 | Ts45 | 0.011167 | 0.002737 |
| *Trachymyrmex septentrionalis* | Ts35 | Ts45 | 0.580569 | 0.025571 |
| *Trachymyrmex septentrionalis* | Ts36 | Ts45 | 1 | 0 |
| *Trachymyrmex septentrionalis* | Ts39 | Ts45 | 0.070138 | 0.015513 |
| *Trachymyrmex septentrionalis* | Ts4 | Ts45 | 0.447843 | 0.021796 |
| *Trachymyrmex septentrionalis* | Ts41 | Ts45 | 0.493452 | 0.028776 |
| *Trachymyrmex septentrionalis* | Ts43 | Ts45 | 0.660766 | 0.030149 |
| *Trachymyrmex septentrionalis* | Ts11 | Ts46 | 1 | 0 |
| *Trachymyrmex septentrionalis* | Ts12 | Ts46 | 0.409498 | 0.016218 |
| *Trachymyrmex septentrionalis* | Ts21 | Ts46 | 1 | 0 |
| *Trachymyrmex septentrionalis* | Ts25 | Ts46 | 1 | 0 |
| *Trachymyrmex septentrionalis* | Ts3 | Ts46 | 1 | 0 |
| *Trachymyrmex septentrionalis* | Ts32 | Ts46 | 1 | 0 |
| *Trachymyrmex septentrionalis* | Ts33 | Ts46 | 0.6822 | 0.029627 |
| *Trachymyrmex septentrionalis* | Ts34 | Ts46 | 0.430012 | 0.01715 |
| *Trachymyrmex septentrionalis* | Ts35 | Ts46 | 1 | 0 |
| *Trachymyrmex septentrionalis* | Ts36 | Ts46 | No information | |
| *Trachymyrmex septentrionalis* | Ts39 | Ts46 | 0.448486 | 0.034185 |
| *Trachymyrmex septentrionalis* | Ts4 | Ts46 | 1 | 0 |
| *Trachymyrmex septentrionalis* | Ts41 | Ts46 | 0.281871 | 0.029027 |
| *Trachymyrmex septentrionalis* | Ts43 | Ts46 | 1 | 0 |
| *Trachymyrmex septentrionalis* | Ts45 | Ts46 | 1 | 0 |
| *Trachymyrmex septentrionalis* | Ts11 | Ts5 | 1 | 0 |
| *Trachymyrmex septentrionalis* | Ts12 | Ts5 | 0.850468 | 0.010752 |
| *Trachymyrmex septentrionalis* | Ts21 | Ts5 | 0.745618 | 0.020617 |
| *Trachymyrmex septentrionalis* | Ts25 | Ts5 | 0.885108 | 0.017523 |
| *Trachymyrmex septentrionalis* | Ts3 | Ts5 | 0.256689 | 0.022087 |
| *Trachymyrmex septentrionalis* | Ts32 | Ts5 | 0.455581 | 0.028815 |
| *Trachymyrmex septentrionalis* | Ts33 | Ts5 | 0.473707 | 0.025218 |
| *Trachymyrmex septentrionalis* | Ts34 | Ts5 | 0.816348 | 0.00823 |
| *Trachymyrmex septentrionalis* | Ts35 | Ts5 | 0.03589 | 0.007321 |
| *Trachymyrmex septentrionalis* | Ts36 | Ts5 | 0.317495 | 0.006862 |
| *Trachymyrmex septentrionalis* | Ts39 | Ts5 | 1 | 0 |
| *Trachymyrmex septentrionalis* | Ts4 | Ts5 | 0* | 0 |
| *Trachymyrmex septentrionalis* | Ts41 | Ts5 | 0.889035 | 0.015363 |
| *Trachymyrmex septentrionalis* | Ts43 | Ts5 | 0.543227 | 0.030454 |
| *Trachymyrmex septentrionalis* | Ts45 | Ts5 | 0.633812 | 0.025766 |
| *Trachymyrmex septentrionalis* | Ts46 | Ts5 | 0.538487 | 0.030478 |
| *Mycetomoellerius turrifex* | Tt5 | Tt10 | 1 | 0 |
| *Mycetomoellerius turrifex* | Tt5 | Tt18 | 1 | 0 |
| *Mycetomoellerius turrifex* | Tt10 | Tt18 | 0.600275 | 0.012249 |
| *Mycetomoellerius turrifex* | Tt5 | Tt20 | 0.120797 | 0.0085 |
| *Mycetomoellerius turrifex* | Tt10 | Tt20 | 1 | 0 |
| *Mycetomoellerius turrifex* | Tt18 | Tt20 | 0.090293 | 0.006622 |
| *Mycetomoellerius turrifex* | Tt5 | Tt2 | 0.199112 | 0.009756 |
| *Mycetomoellerius turrifex* | Tt10 | Tt2 | 0.318433 | 0.013129 |
| *Mycetomoellerius turrifex* | Tt18 | Tt2 | 0.891449 | 0.006289 |
| *Mycetomoellerius turrifex* | Tt20 | Tt2 | 0.477827 | 0.010645 |
| *Mycetomoellerius turrifex* | Tt5 | Tt7 | 0.566671 | 0.003481 |
| *Mycetomoellerius turrifex* | Tt10 | Tt7 | 0.204039 | 0.004282 |
| *Mycetomoellerius turrifex* | Tt18 | Tt7 | 0.782205 | 0.004294 |
| *Mycetomoellerius turrifex* | Tt20 | Tt7 | 0.556096 | 0.004883 |
| *Mycetomoellerius turrifex* | Tt2 | Tt7 | 0.005726 | 0.000644 |
| *Mycetomoellerius turrifex* | Tt5 | Tt9 | 0.081883 | 0.005605 |
| *Mycetomoellerius turrifex* | Tt10 | Tt9 | 0.135604 | 0.006866 |
| *Mycetomoellerius turrifex* | Tt18 | Tt9 | 1 | 0 |
| *Mycetomoellerius turrifex* | Tt20 | Tt9 | 0.062225 | 0.00507 |
| *Mycetomoellerius turrifex* | Tt2 | Tt9 | 1 | 0 |
| *Mycetomoellerius turrifex* | Tt7 | Tt9 | 0.731544 | 0.00451 |
| *Mycetomoellerius turrifex* | Tt5 | Tt16 | 1 | 0 |
| *Mycetomoellerius turrifex* | Tt10 | Tt16 | 1 | 0 |
| *Mycetomoellerius turrifex* | Tt18 | Tt16 | 0.688936 | 0.008382 |
| *Mycetomoellerius turrifex* | Tt20 | Tt16 | 1 | 0 |
| *Mycetomoellerius turrifex* | Tt2 | Tt16 | 0.372037 | 0.008434 |
| *Mycetomoellerius turrifex* | Tt7 | Tt16 | 0.948563 | 0.001395 |
| *Mycetomoellerius turrifex* | Tt9 | Tt16 | 1 | 0 |
| *Mycetomoellerius turrifex* | Tt5 | Tt6 | 1 | 0 |
| *Mycetomoellerius turrifex* | Tt10 | Tt6 | 1 | 0 |
| *Mycetomoellerius turrifex* | Tt18 | Tt6 | 0.72779 | 0.010009 |
| *Mycetomoellerius turrifex* | Tt20 | Tt6 | 1 | 0 |
| *Mycetomoellerius turrifex* | Tt2 | Tt6 | 0.055807 | 0.005095 |
| *Mycetomoellerius turrifex* | Tt7 | Tt6 | 0.133373 | 0.003669 |
| *Mycetomoellerius turrifex* | Tt9 | Tt6 | 1 | 0 |
| *Mycetomoellerius turrifex* | Tt16 | Tt6 | 1 | 0 |
| *Mycetomoellerius turrifex* | Tt5 | Tt14 | 1 | 0 |
| *Mycetomoellerius turrifex* | Tt10 | Tt14 | 1 | 0 |
| *Mycetomoellerius turrifex* | Tt18 | Tt14 | 0.247846 | 0.011589 |
| *Mycetomoellerius turrifex* | Tt20 | Tt14 | 0.401512 | 0.01207 |
| *Mycetomoellerius turrifex* | Tt2 | Tt14 | 0.576994 | 0.010737 |
| *Mycetomoellerius turrifex* | Tt7 | Tt14 | 0.274983 | 0.004651 |
| *Mycetomoellerius turrifex* | Tt9 | Tt14 | 1 | 0 |
| *Mycetomoellerius turrifex* | Tt16 | Tt14 | 0.32622 | 0.009901 |
| *Mycetomoellerius turrifex* | Tt6 | Tt14 | 1 | 0 |
| *Mycetomoellerius turrifex* | Tt5 | Tt15 | 1 | 0 |
| *Mycetomoellerius turrifex* | Tt10 | Tt15 | 1 | 0 |
| *Mycetomoellerius turrifex* | Tt18 | Tt15 | 1 | 0 |
| *Mycetomoellerius turrifex* | Tt20 | Tt15 | 1 | 0 |
| *Mycetomoellerius turrifex* | Tt2 | Tt15 | 1 | 0 |
| *Mycetomoellerius turrifex* | Tt7 | Tt15 | 1 | 0 |
| *Mycetomoellerius turrifex* | Tt9 | Tt15 | No information | |
| *Mycetomoellerius turrifex* | Tt16 | Tt15 | 1 | 0 |
| *Mycetomoellerius turrifex* | Tt6 | Tt15 | 1 | 0 |
| *Mycetomoellerius turrifex* | Tt14 | Tt15 | 1 | 0 |
| *Mycetomoellerius turrifex* | Tt5 | Tt17 | 1 | 0 |
| *Mycetomoellerius turrifex* | Tt10 | Tt17 | 1 | 0 |
| *Mycetomoellerius turrifex* | Tt18 | Tt17 | 1 | 0 |
| *Mycetomoellerius turrifex* | Tt20 | Tt17 | 1 | 0 |
| *Mycetomoellerius turrifex* | Tt2 | Tt17 | 1 | 0 |
| *Mycetomoellerius turrifex* | Tt7 | Tt17 | 0.501938 | 0.005908 |
| *Mycetomoellerius turrifex* | Tt9 | Tt17 | No information | |
| *Mycetomoellerius turrifex* | Tt16 | Tt17 | 1 | 0 |
| *Mycetomoellerius turrifex* | Tt6 | Tt17 | 1 | 0 |
| *Mycetomoellerius turrifex* | Tt14 | Tt17 | 1 | 0 |
| *Mycetomoellerius turrifex* | Tt15 | Tt17 | 0.021284 | 0.005161 |
| *Trachymyrmex arizonensis* | Ts11 | Ts12 | 1 | 0 |
| *Trachymyrmex arizonensis* | Ts11 | Ts33 | 0.49979 | 0.001125 |
| *Trachymyrmex arizonensis* | Ts12 | Ts33 | 0.829596 | 0.001046 |
| *Trachymyrmex arizonensis* | Ts11 | Ts35 | 0.248931 | 0.000824 |
| *Trachymyrmex arizonensis* | Ts12 | Ts35 | 1 | 0 |
| *Trachymyrmex arizonensis* | Ts33 | Ts35 | 0.571038 | 0.001298 |
| *Trachymyrmex arizonensis* | Ts11 | Ts38 | 0.373345 | 0.001715 |
| *Trachymyrmex arizonensis* | Ts12 | Ts38 | 0.870309 | 0.001455 |
| *Trachymyrmex arizonensis* | Ts33 | Ts38 | 0.802506 | 0.00174 |
| *Trachymyrmex arizonensis* | Ts35 | Ts38 | 0.74145 | 0.001802 |
| *Trachymyrmex arizonensis* | Ts11 | Ts39 | 0.499962 | 0.00216 |
| *Trachymyrmex arizonensis* | Ts12 | Ts39 | 0.313893 | 0.002491 |
| *Trachymyrmex arizonensis* | Ts33 | Ts39 | 1 | 0 |
| *Trachymyrmex arizonensis* | Ts35 | Ts39 | 1 | 0 |
| *Trachymyrmex arizonensis* | Ts38 | Ts39 | 0.11007 | 0.003117 |
| *Trachymyrmex arizonensis* | Ts11 | Ts4 | 1 | 0 |
| *Trachymyrmex arizonensis* | Ts12 | Ts4 | 0.48732 | 0.001743 |
| *Trachymyrmex arizonensis* | Ts33 | Ts4 | 0.371869 | 0.001673 |
| *Trachymyrmex arizonensis* | Ts35 | Ts4 | 0.785086 | 0.0014 |
| *Trachymyrmex arizonensis* | Ts38 | Ts4 | 0.585115 | 0.002757 |
| *Trachymyrmex arizonensis* | Ts39 | Ts4 | 1 | 0 |
| *Trachymyrmex arizonensis* | Ts11 | Ts41 | 0.33155 | 0.001164 |
| *Trachymyrmex arizonensis* | Ts12 | Ts41 | 1 | 0 |
| *Trachymyrmex arizonensis* | Ts33 | Ts41 | 0.734932 | 0.001052 |
| *Trachymyrmex arizonensis* | Ts35 | Ts41 | 0.467612 | 0.001999 |
| *Trachymyrmex arizonensis* | Ts38 | Ts41 | 1 | 0 |
| *Trachymyrmex arizonensis* | Ts39 | Ts41 | 0.401951 | 0.003027 |
| *Trachymyrmex arizonensis* | Ts4 | Ts41 | 1 | 0 |
| *Trachymyrmex arizonensis* | Ts11 | Ts44 | 0.62028 | 0.001628 |
| *Trachymyrmex arizonensis* | Ts12 | Ts44 | 0.090054 | 0.001595 |
| *Trachymyrmex arizonensis* | Ts33 | Ts44 | 1 | 0 |
| *Trachymyrmex arizonensis* | Ts35 | Ts44 | 0.743673 | 0.001729 |
| *Trachymyrmex arizonensis* | Ts38 | Ts44 | 0.494046 | 0.004035 |
| *Trachymyrmex arizonensis* | Ts39 | Ts44 | 0.071527 | 0.002613 |
| *Trachymyrmex arizonensis* | Ts4 | Ts44 | 0.588447 | 0.002876 |
| *Trachymyrmex arizonensis* | Ts41 | Ts44 | 1 | 0 |
| *Trachymyrmex arizonensis* | Ts11 | Ts45 | 0.250199 | 0.000668 |
| *Trachymyrmex arizonensis* | Ts12 | Ts45 | 0.571078 | 0.000907 |
| *Trachymyrmex arizonensis* | Ts33 | Ts45 | 1 | 0 |
| *Trachymyrmex arizonensis* | Ts35 | Ts45 | 0.67929 | 0.0008 |
| *Trachymyrmex arizonensis* | Ts38 | Ts45 | 0.356571 | 0.001828 |
| *Trachymyrmex arizonensis* | Ts39 | Ts45 | 0.785139 | 0.001422 |
| *Trachymyrmex arizonensis* | Ts4 | Ts45 | 1 | 0 |
| *Trachymyrmex arizonensis* | Ts41 | Ts45 | 0.734045 | 0.001159 |
| *Trachymyrmex arizonensis* | Ts44 | Ts45 | 1 | 0 |
| *Trachymyrmex pomonae* | Ts11 | Ts13 | 0.164648 | 0.000801 |
| *Trachymyrmex pomonae* | Ts11 | Ts21 | 0.499859 | 0.001709 |
| *Trachymyrmex pomonae* | Ts13 | Ts21 | 0.702647 | 0.002014 |
| *Trachymyrmex pomonae* | Ts11 | Ts32 | 0.499872 | 0.001061 |
| *Trachymyrmex pomonae* | Ts13 | Ts32 | 0.798751 | 0.00131 |
| *Trachymyrmex pomonae* | Ts21 | Ts32 | 1 | 0 |
| *Trachymyrmex pomonae* | Ts11 | Ts35 | 1 | 0 |
| *Trachymyrmex pomonae* | Ts13 | Ts35 | 0.49856 | 0.001043 |
| *Trachymyrmex pomonae* | Ts21 | Ts35 | 1 | 0 |
| *Trachymyrmex pomonae* | Ts32 | Ts35 | 0.167347 | 0.000731 |
| *Trachymyrmex pomonae* | Ts11 | Ts38 | 1 | 0 |
| *Trachymyrmex pomonae* | Ts13 | Ts38 | 0.602768 | 0.001341 |
| *Trachymyrmex pomonae* | Ts21 | Ts38 | 1 | 0 |
| *Trachymyrmex pomonae* | Ts32 | Ts38 | 0.599252 | 0.00136 |
| *Trachymyrmex pomonae* | Ts35 | Ts38 | 1 | 0 |
| *Trachymyrmex pomonae* | Ts11 | Ts39 | 1 | 0 |
| *Trachymyrmex pomonae* | Ts13 | Ts39 | 1 | 0 |
| *Trachymyrmex pomonae* | Ts21 | Ts39 | 0.795013 | 0.001878 |
| *Trachymyrmex pomonae* | Ts32 | Ts39 | 1 | 0 |
| *Trachymyrmex pomonae* | Ts35 | Ts39 | 1 | 0 |
| *Trachymyrmex pomonae* | Ts38 | Ts39 | 0.199619 | 0.001018 |
| *Trachymyrmex pomonae* | Ts11 | Ts41 | 1 | 0 |
| *Trachymyrmex pomonae* | Ts13 | Ts41 | 1 | 0 |
| *Trachymyrmex pomonae* | Ts21 | Ts41 | 1 | 0 |
| *Trachymyrmex pomonae* | Ts32 | Ts41 | 0.20089 | 0.000821 |
| *Trachymyrmex pomonae* | Ts35 | Ts41 | 1 | 0 |
| *Trachymyrmex pomonae* | Ts38 | Ts41 | 0.199897 | 0.000732 |
| *Trachymyrmex pomonae* | Ts39 | Ts41 | 1 | 0 |
| *Trachymyrmex pomonae* | Ts11 | Ts44 | 1 | 0 |
| *Trachymyrmex pomonae* | Ts13 | Ts44 | 1 | 0 |
| *Trachymyrmex pomonae* | Ts21 | Ts44 | 0.798987 | 0.00191 |
| *Trachymyrmex pomonae* | Ts32 | Ts44 | 1 | 0 |
| *Trachymyrmex pomonae* | Ts35 | Ts44 | 1 | 0 |
| *Trachymyrmex pomonae* | Ts38 | Ts44 | 1 | 0 |
| *Trachymyrmex pomonae* | Ts39 | Ts44 | 0.599594 | 0.00201 |
| *Trachymyrmex pomonae* | Ts41 | Ts44 | 0.399419 | 0.000576 |
| *Trachymyrmex pomonae* | Ts11 | Ts45 | 0.333656 | 0.001234 |
| *Trachymyrmex pomonae* | Ts13 | Ts45 | 0.401851 | 0.001611 |
| *Trachymyrmex pomonae* | Ts21 | Ts45 | 0.799125 | 0.001829 |
| *Trachymyrmex pomonae* | Ts32 | Ts45 | 0.200451 | 0.001579 |
| *Trachymyrmex pomonae* | Ts35 | Ts45 | 1 | 0 |
| *Trachymyrmex pomonae* | Ts38 | Ts45 | 1 | 0 |
| *Trachymyrmex pomonae* | Ts39 | Ts45 | 0.601775 | 0.001962 |
| *Trachymyrmex pomonae* | Ts41 | Ts45 | 1 | 0 |
| *Trachymyrmex pomonae* | Ts44 | Ts45 | 1 | 0 |
| *Trachymyrmex pomonae* | Ts11 | Ts46 | 1 | 0 |
| *Trachymyrmex pomonae* | Ts13 | Ts46 | 1 | 0 |
| *Trachymyrmex pomonae* | Ts21 | Ts46 | 1 | 0 |
| *Trachymyrmex pomonae* | Ts32 | Ts46 | 0.466853 | 0.002221 |
| *Trachymyrmex pomonae* | Ts35 | Ts46 | 1 | 0 |
| *Trachymyrmex pomonae* | Ts38 | Ts46 | 0.331316 | 0.001737 |
| *Trachymyrmex pomonae* | Ts39 | Ts46 | 0.730592 | 0.001749 |
| *Trachymyrmex pomonae* | Ts41 | Ts46 | 0.399702 | 0.001129 |
| *Trachymyrmex pomonae* | Ts44 | Ts46 | 0.73523 | 0.001852 |
| *Trachymyrmex pomonae* | Ts45 | Ts46 | 0.73315 | 0.001912 |
| *Trachymyrmex pomonae* | Ts11 | Ts7 | 1 | 0 |
| *Trachymyrmex pomonae* | Ts13 | Ts7 | 1 | 0 |
| *Trachymyrmex pomonae* | Ts21 | Ts7 | 0.500159 | 0.00153 |
| *Trachymyrmex pomonae* | Ts32 | Ts7 | 1 | 0 |
| *Trachymyrmex pomonae* | Ts35 | Ts7 | 1 | 0 |
| *Trachymyrmex pomonae* | Ts38 | Ts7 | 1 | 0 |
| *Trachymyrmex pomonae* | Ts39 | Ts7 | 1 | 0 |
| *Trachymyrmex pomonae* | Ts41 | Ts7 | 0.400771 | 0.000543 |
| *Trachymyrmex pomonae* | Ts44 | Ts7 | 1 | 0 |
| *Trachymyrmex pomonae* | Ts45 | Ts7 | 1 | 0 |
| *Trachymyrmex pomonae* | Ts46 | Ts7 | 1 | 0 |
