# Supplementary material for: Development, characterization, and cross-amplification of polymorphic microsatellite markers for North American Trachymyrmex and Mycetomoellerius ants
Source: BMC Res Notes. 2020 Mar 24;13:173. doi: 10.1186/s13104-020-05015-3 (PMC7092486; doi:10.1186/s13104-020-05015-3)
Supplement: Supplementary file 5 — Additional file 5. Details of the 17 Trachymyrmex septentrionalis polymorphic microsatellite markers analyzed for Trachymyrmex septentrionalis ants across Texas (TX) and Florida (FL), highlighting the differences between two distant populations. Details include: marker name, size range of observed alleles given in base pairs (bp), number of individuals genotyped (N), number of alleles observed (K). [file 13104_2020_5015_MOESM5_ESM.docx]

Additional file 5. Details of the 17 *Trachymyrmex septentrionalis* polymorphic microsatellite markers analyzed for *Trachymyrmex septentrionalis* ants across Texas (TX) and Florida (FL), highlighting the differences between two distant populations. Details include: marker name, size range of observed alleles given in base pairs (bp), number of individuals genotyped (N), number of alleles observed (K).

|  | TX | | | FL | | |
| --- | --- | --- | --- | --- | --- | --- |
| Marker | **Size (bp)** | **N** | **K** | **Size (bp)** | **N** | **K** |
| Ts3 | 288-294 | 3 | 2 | 264-292 | 25 | 12 |
| Ts4 | 220-260 | 4 | 3 | 242-266 | 25 | 11 |
| Ts5 | 206-248 | 4 | 3 | 232-262 | 25 | 11 |
| Ts11 | 270-358 | 4 | 6 | 274-353 | 20 | 13 |
| Ts12 | 158-174 | 4 | 3 | 164-214 | 26 | 8 |
| Ts21 | 314-322 | 4 | 2 | 302-326 | 26 | 10 |
| Ts25 | 252-312 | 4 | 4 | 286-346 | 26 | 16 |
| Ts32 | 228-242 | 3 | 4 | 200-242 | 25 | 10 |
| Ts33 | 272-284 | 4 | 3 | 260-296 | 26 | 12 |
| Ts34 | 394-426 | 3 | 2 | 246-490 | 26 | 11 |
| Ts35 | 284-304 | 4 | 4 | 280-306 | 27 | 10 |
| Ts36 | 192-228 | 4 | 5 | 166-210 | 13 | 9 |
| Ts39 | 238-254 | 4 | 4 | 242-326 | 27 | 18 |
| Ts41 | 212-220 | 4 | 4 | 206-236 | 27 | 12 |
| Ts43 | 282-304 | 4 | 2 | 276-352 | 25 | 24 |
| Ts45 | 198-216 | 3 | 4 | 174-274 | 25 | 16 |
| Ts46 | 314-330 | 4 | 2 | 314-348 | 25 | 19 |
